# Measuring Scale Errors in a Laser Tracker’s Horizontal Angle Encoder Through Simple Length Measurement and Two-Face System Tests

**DOI:** 10.6028/jres.115.022

**Published:** 2010-10-01

**Authors:** B. Muralikrishnan, C. Blackburn, D. Sawyer, S. Phillips, R. Bridges

**Affiliations:** Precision Engineering Division, National Institute of Standards and Technology, Gaithersburg, MD 20899; FARO Technologies, Kennett Square, PA 19348

**Keywords:** ASME B89.4.19, encoder scale error, geometric misalignment, laser tracker, second order harmonic

## Abstract

We describe a method to estimate the scale errors in the horizontal angle encoder of a laser tracker in this paper. The method does not require expensive instrumentation such as a rotary stage or even a calibrated artifact. An uncalibrated but stable length is realized between two targets mounted on stands that are at tracker height. The tracker measures the distance between these two targets from different azimuthal positions (say, in intervals of 20° over 360°). Each target is measured in both front face and back face. Low order harmonic scale errors can be estimated from this data and may then be used to correct the encoder’s error map to improve the tracker’s angle measurement accuracy. We have demonstrated this for the second order harmonic in this paper. It is important to compensate for even order harmonics as their influence cannot be removed by averaging front face and back face measurements whereas odd orders can be removed by averaging. We tested six trackers from three different manufacturers. Two of those trackers are newer models introduced at the time of writing of this paper. For older trackers from two manufacturers, the length errors in a 7.75 m horizontal length placed 7 m away from a tracker were of the order of ± 65 μm before correcting the error map. They reduced to less than ± 25 μm after correcting the error map for second order scale errors. Newer trackers from the same manufacturers did not show this error. An older tracker from a third manufacturer also did not show this error.

## 1. Introduction

The spherical coordinates of a target (horizontal angle, vertical angle and range) reported by a laser tracker suffer from systematic errors due to various geometric and optical misalignments [1]. Some misalignments such as encoder eccentricity, beam offset, beam tilt, etc., are modeled and corrected for in the software. In this paper, we discuss an error source, the scale error in the horizontal angle encoder of the tracker, which is generally not considered during error modeling and compensation, but may potentially produce large errors in long length measurements made far away from the tracker.

The scale errors in the encoder are spacing errors in the gratings (or markings). In Coordinate Measuring Machines, scale errors are one source of linear displacement errors and can be calibrated using a laser interferometer. But an analogous technique to calibrate the angle encoder of a laser tracker is somewhat cumbersome and requires expensive and highly accurate instrumentation. For example, Gassner and Ruland [2] have performed a calibration of the angle encoder of their laser tracker using a calibrated rotary table whose accuracy exceeds that of the encoder under test. They report angle errors of about 20 μrad peak-to-valley on a laser tracker. They further report that the second order harmonic is the dominant frequency in the error plot of their encoder. Ouyang et al., [3] have attempted to calibrate the angle errors in a tracker by mounting the tracker on a CMM and performing circle measurements in different planes.

As opposed to the direct methods for angle error estimation mentioned above, we propose an indirect method where we determine the parameters of a model for scale errors. The key advantage is the simplicity of the technique; we do not require expensive instrumentation such as a calibrated rotary table or even a calibrated reference length. On the other hand, our method does not provide a direct error map of the scale errors. But it is a method that can be implemented in any laboratory with reasonable degree of environmental control to obtain a fairly good estimate of the quality of the angle encoder in a tracker. Further, the method is very closely related to tests described in the ASME B89.4.19 Standard [4] and therefore manufacturers and users may already have necessary experience and materials to perform the test.

## 2. Approach

The errors in the scale *e* of the angle encoder may be decomposed into their Fourier components. The larger amplitudes are generally associated with low order harmonics, as the Gassner and Ruland [2] results suggest, and therefore higher order harmonics may be disregarded from consideration. Approximated as the sum of the first *n* harmonics, *e* is given by

(1) $$e(\phi )\approx \sum_{i=1}^{n}a_{i}\;\cos(i\phi )+\sum_{i=1}^{n}b_{i}\;\sin(i\phi )$$

where *a_i_* and *b_i_* are the amplitudes of the *i*th harmonic, and *ϕ* is the measured horizontal (azimuth) angle. The true angle *ϕ_t_*, assuming other sources of errors are absent, can then be given by: *ϕ_t_* =*ϕ* − *e* (*ϕ*).

### Detecting Odd Orders From Two-Face Tests

If the scale error contains only odd order harmonics, the error in the measured horizontal angle in the front face is equal in magnitude but opposite in sign to that measured in the back face. A two-face system test (the apparent distance between measurements of a stationary target in front face and then in back face) as described in [4] therefore produces a non-negative error (or apparent distance) in this case.

This sensitivity to front-face back-face measurements may, in principle, be exploited to determine the parameters (*a_i_*, *b_i_*) for odd orders in Eq. (1). That is, half the difference between front-face azimuth measurements and back-face azimuth measurements to a stationary target placed at different azimuthal positions *ϕ* yields errors *e*(*ϕ*) that may be used to determine parameters *a_i_* and *b_i_* for odd orders. It should be noted that in addition to being sensitive to odd order harmonics, such front-face back-face measurements are also sensitive to numerous other sources of misalignment such as collimation error, non-orthogonality between the standing axis and the transit axis, etc. The other sources generally produce errors that are constant (because vertical angle does not change between measurements performed at different azimuths); therefore residuals from the mean of half the difference between front face and back face measurements are to be considered when determining odd orders from Eq. (1).

It should be pointed out however that estimating odd order harmonics is not critical because their effect can be removed by averaging between front face and back face measurements. We therefore do not consider odd orders in detail in this paper.

### Detecting Even Orders

If the scale error contained purely even order terms (*n* = 2, 4, 6,…), the two-face test [4] produces a result of zero because the error in the measured horizontal angle at *ϕ* is identical in magnitude and sign to the error at *ϕ* + 180°. Even order harmonics are therefore not sensitive to two-face tests.

It is therefore proposed to use simple length measurement system tests to determine parameters of the model in Eq. (1), for even order harmonics. The position and orientation of the length has to be chosen carefully so that it is sensitive primarily to encoder scale errors, and not to other geometric errors that may be a function of range or vertical angle.

Estimating low even order harmonics is critical because, if present, they cannot be removed by averaging front and back face measurements as mentioned earlier. The encoder’s error map has to be compensated for even order harmonics to enhance the tracker’s accuracy.

### Experimental Setup

**Fig. 1:**
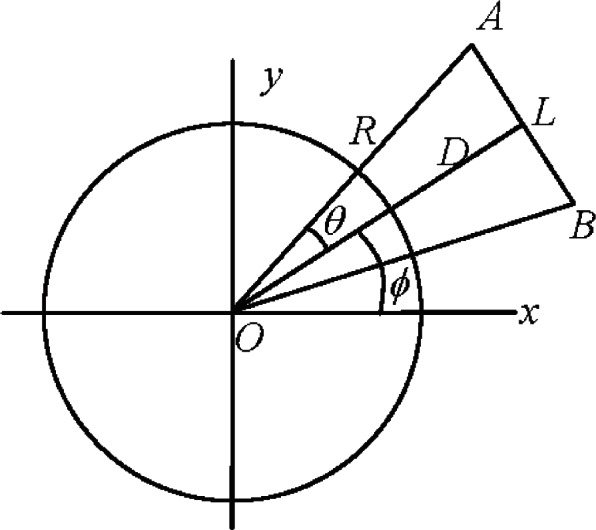
Reference *AB* of length *L* positioned distance *D* from the tracker located at *O*. The range to the targets at *A* and *B* is *R*. The reference length *L* subtends an angle 2*θ* at the origin *O* and the bisector of *L* is located at an angle *ϕ* from the *x* axis.

A horizontal length *L* symmetrically placed with respect to the tracker and positioned at roughly tracker height (as described in the ASME B89.4.19 Standard) is ideally suited for this purpose (see Fig. 1). Because the range and the vertical angle to the two targets are equal, we eliminate any sources that may produce horizontal angle errors that are functions of nominal range and nominal vertical angle (for example, those arising from a squareness error, collimation error, beam offset along the transit axis, etc.). On the other hand, the test is sensitive to those sources that produce horizontal angle errors that are a function of the nominal horizontal angle, such as encoder scale errors and eccentricity of the encoder with respect to the vertical axis. Encoder eccentricity is a first order harmonic and is usually already compensated for in the software, leaving only higher order harmonics which are due to scale errors.

The test involves measuring a fixed length placed at different azimuthal positions of the tracker, in a manner similar to the horizontal length tests in the ASME B89.4.19 Standard. As a matter of practical convenience, we realize the length not by using a physical artifact, but as the distance between two targets on stands. After measuring the distance between the targets using the tracker, we rotate the tracker about its vertical axis and re-measure the distance between the targets. We repeat this process several times to cover a full circle (360° of the encoder). It is not necessary to take special care when performing this rotation. The mathematics involved in determining the scale error incorporates the actual range and azimuths as measured to the targets.

**Fig. 2:**
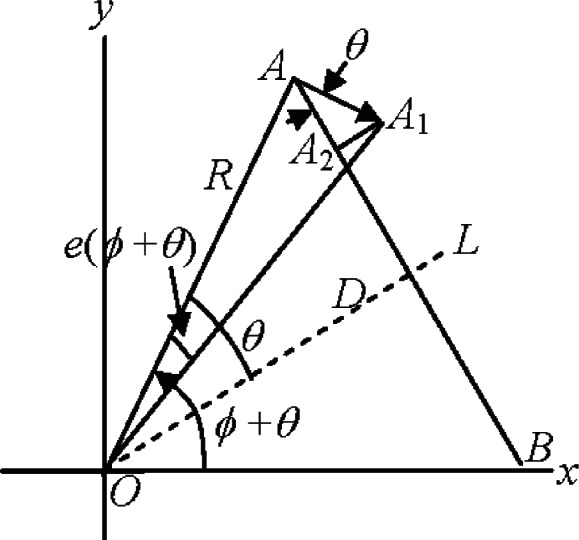
Calculating the error in the length *AB* due to scale error in the encoder.

The error *E* in the measured length *L* can be calculated from some simple trigonometry as shown, see Fig. 2.

The angle *θ* is half the angle subtended by the reference length *AB* at the tracker, *D* is the distance from the tracker to the length *AB*, *R* is the range to the target, and *ϕ* is the azimuthal position of the tracker as measured to the center of the length *AB* (see Fig. 1 and Fig. 2). The azimuthal position of target *A* is *ϕ* + *θ*. The scale error at *A* is *e* (*ϕ* + *θ*). The range to target *A* is *R*. According to the adopted convention, a positive scale error implies the measured angle is larger than the true angle. We assume that *e* (*ϕ* + *θ*) is positive and therefore the measured angle is larger than the true angle. Therefore, the true position of point *A* is at *A*_1_, where the azimuth is *ϕ* + *θ* − *e* (*ϕ* + *θ*). *AA*_1_ is the error vector. Its magnitude is *Re* (*ϕ* + *θ*). The component of the error along the length *AA*_2_ is given by *AA*_1_cos(*θ*) = *Re* (*ϕ* + *θ*)cos(*θ*). The difference in the errors at *A* and *B* (the azimuth at *B* is *ϕ* – *θ*) is the net error in the length and is given by

(2) E(θ,ϕ)=Re(ϕ+θ)cosθ−Re(ϕ−θ)cosθ.

For the purposes of this experiment, the distance between the targets does not have to be calibrated by other means; it is only required that the distance not change during the duration of the experiments. We consider the *residuals from the average of the multiple length measurements at different azimuths ϕ as the errors* due to scale, *E*, in Eq. (2) above. It should be noted that there may be other sources of systematic error such as, for instance, a ranging error in the tracker that will remain identical for all the lengths measured. These systematic sources produce a bias (from the true but unknown length between *A* and *B*) which is removed by discarding the mean of all the measured lengths and considering only the residuals. We can then fit Eq. (2) to the residuals to determine parameters (*a_i_*, *b_i_*) for both even and odd harmonics. It should be noted that systematic errors in the ranging system are detected by a separate ranging test [4]. We also point out that there may other sources of error, such as a cyclical error in range that may not be identical for all measured lengths, and must be accounted for in the uncertainty budget.

**Fig. 3:**
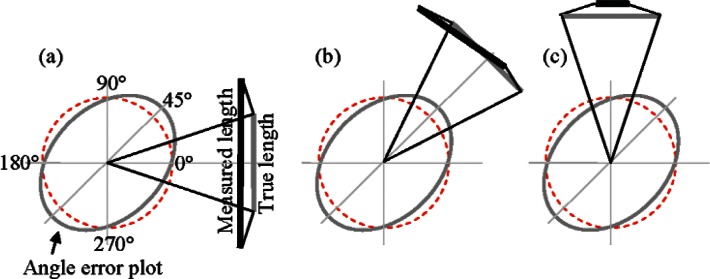
Second order scale errors in the encoder (sin(2*ϕ*) term only for purposes of illustration) are shown in the figure. (a) The errors in the angle at the two ends are such that the measured length is larger than the true length (in our assumed sign convention where positive error in angle represents larger measured angle in comparison to truth). (b) The errors in the angle at the two ends of the length only serve to rotate the length but not change its magnitude. (c) The errors in the angle at the two ends of the length are such that the measured length is smaller than the true length. Thus, the errors in the length of the reference artifact will also show a dominant second order harmonic if the scale error has a dominant second order harmonic.

We note that the purpose of correcting scale errors is to improve the accuracy of the tracker, and that typically, high accuracy measurements involve averaging front face and back face measurements at every target position. Averaging front and back face measurements, as mentioned earlier, removes the influence of several geometric misalignments including odd order harmonics in the scale error. We therefore adopt this approach in our experiments also, hence averaging front and back face measurements means that we only attempt to fit even order harmonics to Eq. (2). As mentioned earlier, we can always assess the presence of odd order harmonics from difference of front face and back face measurements made at the different azimuths. Fig. 3 illustrates why the horizontal length test is sensitive to second order scale error in the encoder.

## 3. Sensitivity Analysis for Second Order Harmonic

Because the second order harmonic is expected to be the dominant error source, we take a closer look at optimizing the test variables (*D* and *L*) to obtain maximum sensitivity to the second order harmonic. We briefly address the issue of determining the length *L* between targets *A* and *B*, and distance *D* from the tracker to obtain maximum sensitivity to second order harmonic scale error in the encoder (see Fig. 1).

The error *E* in the measured length *L* due to a second order harmonic scale error can be given by

(3) $$\begin{array}{l} E(\theta ,\phi )=R\;\cos\;\theta [e(\phi +\theta )-e(\phi -\theta )]\\ =R\;\cos\;\theta [a_{2}\;\cos\;(2(\phi +\theta ))+b_{2}\;\sin(2(\phi +\theta ))\\ \;-a_{2}\;\cos(2(\phi -\theta ))-b_{2}\;\sin(2(\phi -\theta ))]. \end{array}$$

Eq. (3) can be simplified to

(4) $$E(\theta ,\phi )=2R\;\cos\;\theta \;\sin\;2\theta \sqrt{a_{2}^{2}+b_{2}^{2}}\sin(\chi -2\phi )$$

where

$$\chi =\sin^{-1}(\frac{b_{2}}{\sqrt{a_{2}^{2}+b_{2}^{2}}}).$$

For any given length *L* and distance *D*, *E* is maximum when sin(*χ* – 2*ϕ*) = 1. The azimuthal location *ϕ* where *E* is maximum can therefore be determined from the above condition if *a*_2_ and *b*_2_ are known.

Our objective here is not only to determine where the maximum error occurs, but more importantly, to determine optimal values for *D* and *L* so that the error at this angular position is as large as possible to achieve maximum sensitivity. Thus, the objective is to maximize the function *f* = 2*R*cos*θ* sin2*θ*, which represents the sensitivity of the measurement, i.e., the length error in micrometers for 1 μrad of second order scale error.

Expressing *f* in terms of the quantities of interest, we have

(5) $$f=2R\;\cos\;\theta \;\sin\;2\theta =\frac{2D^{2}L}{D^{2}+\frac{L^{2}}{4}}.$$

**Fig. 4:**
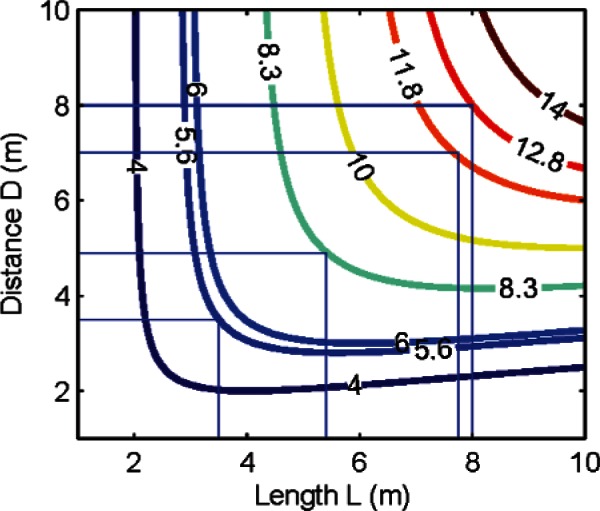
Contour plot showing sensitivity (function value *f* in units of micrometers of length error per one microradian of second order harmonic in the scale error) as a function of *D* and *L*.

We plot *f* as a function of *D* and *L* in the contour plot shown in Fig. 4. Clearly, a large length *L* placed far away from the tracker provides maximum sensitivity. For example, the maximum error in the length for 1 μrad of second order scale error is 5.6 μm for a 3.5 m length placed 3.5 m away, but increases to 12.8 μm for an 8 m length placed 8 m away.

The above sensitivity analysis does not however account for the decreasing accuracy in tracker measurements made farther away or for the effect of variations in the distance between the two targets due to short term thermal fluctuations. We address the issue of uncertainty in the calculated parameters *a*_2_ and *b*_2_, and in the resulting error map after we present the experimental results.

## 4. Experimental Results

We performed the length measurement system tests as described in Sec. 2 on six trackers from three different manufacturers and also at different combinations of *L* and *D*. Trackers A and B are older models and E is a newer model from one manufacturer. Tracker C is an older model and F is a newer model from a second manufacturer. Tracker D is an older model from a third manufacturer. The different combinations of *L* and *D* were chosen to assess the validity of the analysis discussed earlier. We present the results here.

### Tracker A

**Fig. 5:**
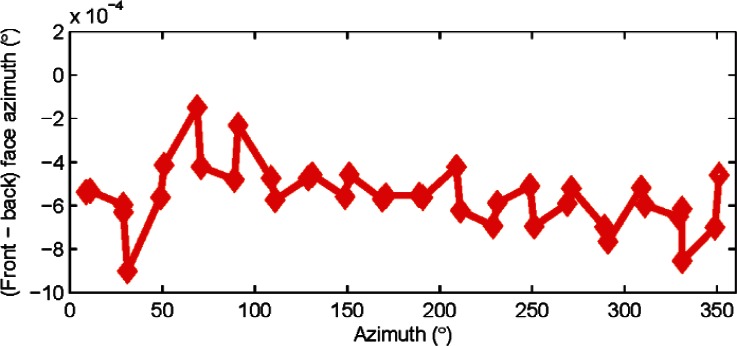
Difference between front and back face azimuth measurements to a target plotted as a function of azimuth. There is no evidence of low odd order harmonics. Data was collected from tracker A.

As mentioned earlier, we measured each target in front and back face. We show a plot of the difference between front and back face azimuth measurements as a function of azimuthal position in Fig. 5. There is no evidence of any low order odd harmonics in the plot. We do not therefore consider mapping odd orders here. Also, as mentioned earlier, any odd order errors in Fig. 5 can be removed by averaging front and back face measurements. We therefore focus on low even orders next.

**Fig. 6:**
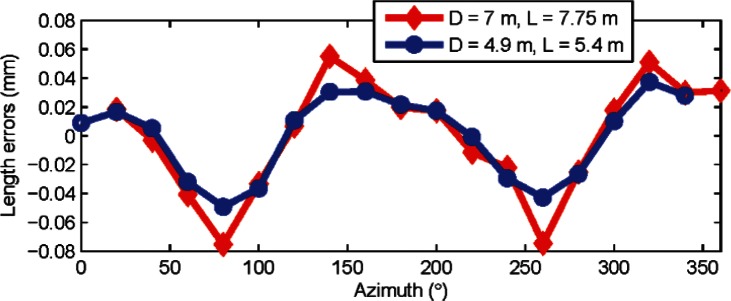
Length errors (residuals from the mean length) are shown in the figure for horizontal length tests for two different combinations (*D* = 7 m, *L* = 7.75 m and *D* = 4.9 m, *L* = 5.4 m) with azimuths taken from the original encoder map of the tracker (i.e., before correcting the tracker map for second order errors). Data was collected from tracker A.

Figure 6 shows the length errors (residues) as a function of azimuth for two combinations of *L* and *D*. The length errors were calculated after averaging front face and back face measurements at each target position, thereby removing the influence of any odd order harmonics.

The dominant harmonic appears to be the second order from Fig. 6. A least-squares best-fit yielded an amplitude of 4.3 μrad $\left(\sqrt{a_{2}^{2}+b_{2}^{2}}\right)$ for the second order harmonic for combination 1 (*D* = 7 m, *L* = 7.75 m) and 4.1 μrad for combination 2 (*D* = 4.9 m, *L* = 5.4 m). This agreement of the second order harmonic to within 0.2 μrad is within its uncertainty as shown in the next section.

As a quick note, we point out that some simple calculations validate the analysis described in the previous section. The sensitivity *f* for combination 1 (*D* = 7 m, *L* = 7.75 m) is 11.8 μm/μrad. The sensitivity for combination 2 (*D* = 4.9 m, *L* = 5.4 m) is 8.3 μm/μrad. The errors (residuals from the mean) in the lengths are within ±65 μm for combination 1 from Fig. 6, and within ± 43 μm for combination 2. The ratio of the maximum error to the sensitivity provides an estimate of the amplitude of second order harmonic scale error, and should be nearly the same regardless of the combination of *L* and *D* used. This of course assumes that the second order is the dominant term, as is the case here. For combination 1, that ratio is 65/11.8 = 5.5 μrad. For combination 2, it is 43/8.3 = 5.2 μrad. They agree fairly well, as expected. There is however some discrepancy between these estimates and the 4.3 μrad (or the 4.1 μrad) value calculated earlier because there are some other higher order even harmonics in the data as well.

**Fig. 7:**
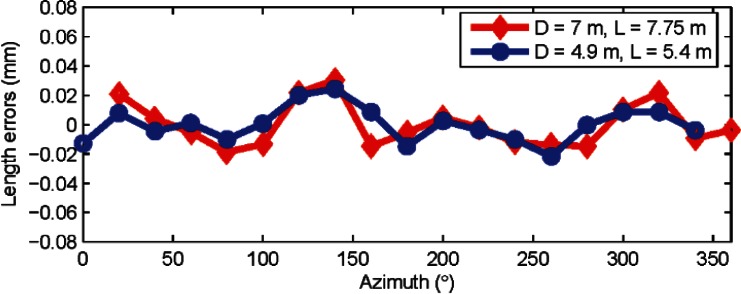
Length errors (residuals from the mean length) are shown in the figure for horizontal length tests for two different combinations (*D* = 7 m, *L* = 7.75 m and *D* = 4.9 m, *L* = 5.4 m) after correcting the tracker map for second order errors in the scale. Data was collected from tracker A.

A rigorous approach to the task of validating our claims would be to correct the error map of our tracker and perform the experiments again; we have in fact done so and the results of our experiments after correcting the tracker’s azimuth encoder map are shown in Fig. 7. It is clear that there is a very small, if any, second order component in the length errors. There is substantial reduction in errors after correcting the error map; the errors are less than ± 25 μm by removing just the second order alone.

The benefits of such second order correction are more clearly observed when measuring large lengths far away from the tracker. A further correction of other even order harmonics may also be performed, but we have not done so with our tracker because the amplitude of these higher orders are almost near the uncertainty achievable with our method. We should point out that downloading the error map from trackers, correcting it, and uploading the new map, while not very challenging, may require special support from the manufacturer of the tracker.

### Tracker B

Tracker B is also an older model from the same manufacturer as tracker A. This tracker also displayed the second order error characteristic as described earlier for tracker A, but with smaller amplitude of about 2.5 μrad.

### Tracker C

**Fig. 8:**
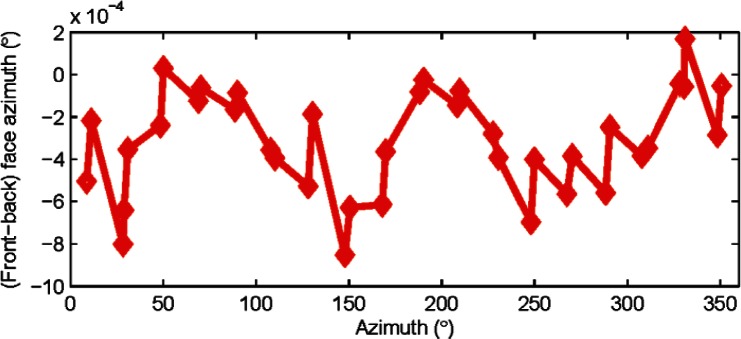
Difference between front and back face azimuth measurements to a target plotted as a function of azimuth. There is no evidence of low odd order harmonics. Data was collected from tracker C.

Tracker C is an older model from a second manufacturer. Fig. 8 shows the difference between front and back face azimuth measurements to a target as a function of azimuth. Low odd orders, if present, are small in magnitude. As mentioned earlier, we do not compensate for odd orders; we simply average front and back face measurements and remove any odd orders.

**Fig. 9:**
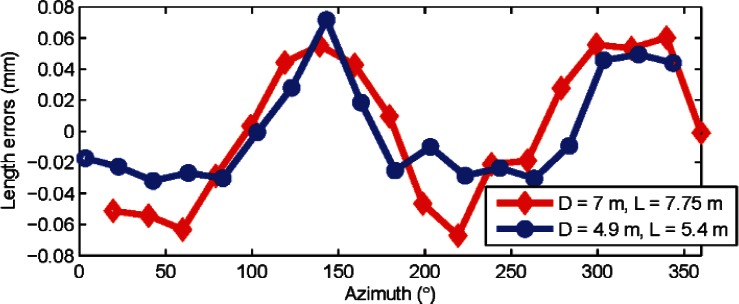
Length errors (residuals from the mean length) are shown in the figure for horizontal length tests for two different combinations (*D* = 7 m, *L* = 7.75 m and *D* = 4.9 m, *L* = 5.4 m) with the original encoder map of the tracker (i.e., before correcting the tracker map for second order errors). Data was collected from tracker C.

Figure 9 shows the results from length tests performed for the two combinations discussed earlier. The dominant harmonic appears to be the second order. A least-squares best-fit yielded an amplitude of 5.0 μrad $\left(\sqrt{a_{2}^{2}+b_{2}^{2}}\right)$ for the second order harmonic for combination 1 (*D* = 7 m, *L* = 7.75 m) and 4.8 μrad for combination 2 (*D* = 4.9 m, *L* = 5.4 m). This agreement of the second order harmonic to within 0.2 μrad is within its uncertainty as shown in the next section.

**Fig. 10:**
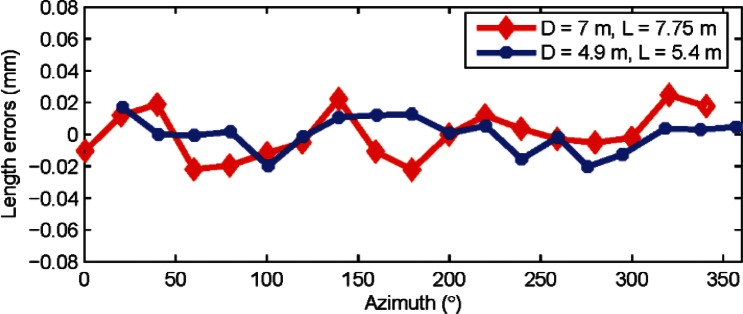
Length errors (residuals from the mean length) are shown in the figure for horizontal length tests for two different combinations (*D* = 7 m, *L* = 7.75 m and *D* = 4.9 m, *L* = 5.4 m) after correcting the tracker map for second order errors in the scale. Data was collected from tracker C.

Fig. 10 shows the results from the length tests after correcting the tracker’s error map for second order errors. There is substantial reduction in errors after correcting the error map; the errors are less than ±25 μm by removing just the second order alone.

### Tracker D

The amplitude of the second order was about 1 μrad for this old tracker from a third manufacturer, much smaller than that seen for the other two trackers. The horizontal length tests however showed errors of about ± 40 μm, possibly because of higher order harmonics.

## 5. Discussion

Low order errors can be removed in several different ways. Odd orders may be removed by having two read-heads positioned 180° apart on the encoder. Even orders may be removed by having two readheads 90° apart. The cost and complexity of having additional readheads may be mitigated by a calibration of the encoder through independent means such as by using a high accuracy rotary stage, a polygon with autocollimator etc. The error mapping technique we have discussed in this paper is another approach for the same.

**Fig. 11:**
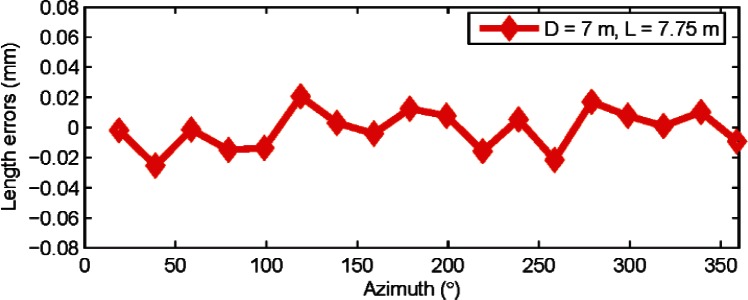
Length errors (residuals from the mean length) are shown in the figure for horizontal length tests on a new tracker design (tracker E) that intrinsically compensates for second order scale errors. As expected, no second order scale error is noticeable.

**Fig. 12:**
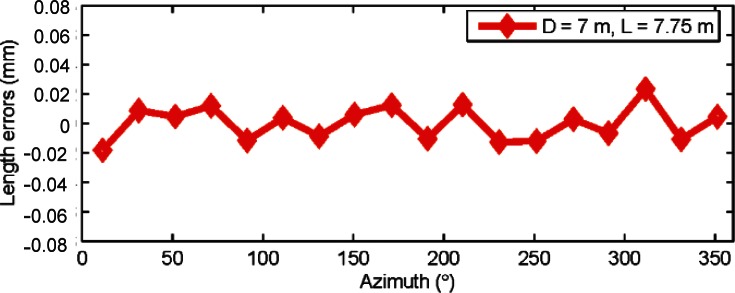
Length errors (residuals from the mean length) are shown in the figure for horizontal length tests for tracker F. No second order scale error is noticeable.

We have tested new tracker designs from two manufacturers, trackers E and F. The results of the horizontal length tests are shown in Fig. 11 for tracker E that intrinsically compensates for second order errors. The errors are much smaller than those in Fig. 6 and, as the manufacturer expected, no second order error is noticeable. We also tested a new tracker (Tracker F) from another manufacturer; the results are shown in Fig. 12. There is no evidence of second order errors.

## 6. A Note on Higher Order Harmonics

The preceding sections described low order harmonic errors in the scale and a simple technique to estimate those errors. There is an analogous effect at the high frequency end where periodic errors have been observed at a frequency representing the spacing between lines in an encoder. We do not directly address compensating high frequency encoder errors in this paper, but point out that a scheme similar to our proposed approach may be considered *for testing purposes* where an uncalibrated horizontal length can be measured by a tracker at closely spaced azimuthal positions so that multiple sampling positions are contained between two adjacent encoder lines. Two-face tests may also be performed so that multiple sampling positions are contained between two adjacent encoder lines. Such tests may reveal interpolation errors in the encoder.

## 7. Uncertainty Considerations

There is an uncertainty associated with each of the lengths that are measured as part of the test described in Sec. 2, and therefore an uncertainty in the parameters *a*_2_ and *b*_2._ We address the issue of determining the uncertainty in these parameters here. Values are calculated for combination 1 (*D* = 7 m, *L* = 7.75 m; the range to targets *R* is 8 m and the half angle *θ* subtended by the length is 29°).

The tracker’s ranging accuracy deteriorates farther away from the tracker. Manufacturers typically specify an MPE (maximum permissible error) for the range (for example, one of the trackers we tested had an MPE specification of 20 μm + 0.8 μm/m). Because the length measurements are always performed so that the target is at the same range (about 8 m) from the tracker, a large portion of the error in the range is expected to be due to systematic sources which produce the same errors in length for all horizontal length measurements. We only consider residuals from the mean of all length measurements. Therefore the error in the range due to systematic sources is not of any consequence, and using the MPE for estimating uncertainty is not appropriate for this type of measurement. On the other hand, the variation in repeated range measurements (i.e., the repeatability) is the noise in the measurement and therefore is the primary source of error that is relevant in our case. We experimentally determine the standard uncertainty due to repeatability in range as 1.5 μm for one of the trackers we tested. Only the component of the ranging uncertainty along the length will actually contribute to a length error. Further, the length is the difference in measurements made at two targets and we average 2 measurements at each target location. Therefore the standard uncertainty in the measured length due to ranging error non-repeatability in the tracker is 1.5 sin*θ* = 0.7 μm.

Manufacturers also sometimes specify an MPE for transverse measurements (for example, one of the trackers we tested had an MPE specification of 36 μm + 6 μm/m for angle measurements). As discussed in the previous paragraph, this MPE incorporates all sources that produce errors in the measured angle, including the second order harmonic scale error in the encoder. Further, this MPE is applicable for measurements made in the front face of the tracker, whereas all our measurements are performed in both faces and averaged. Therefore, using the MPE as a bound on the uncertainty is not appropriate for this type of measurement. Because it is the purpose of our experiments to determine the extent of one source of systematic error—namely the second order harmonic, it is necessary to primarily assess the repeatability of the angle measurement system which introduces noise into the measurement. We experimentally measured the standard uncertainty due to repeatability in the horizontal angle to be 0.0001° for one of the trackers we tested. At a range of 8 m, considering 2 targets, 2 repeat measurements at each target, and only the component along the length, the standard uncertainty in length due to repeatability in the measured horizontal angle is 8 (0.0001*π*/180)cos*θ* = 12.2 μm.

As long lengths are considered in the experiments, stability of the length (realized between two targets on stands) during the duration of the experiment (about 1 hour) is important. To estimate the stability of the length, we mount an interferometer on one stand and a target on the other, and record the length in intervals of 5 seconds over a one hour period. The standard uncertainty in the measured lengths is about 1.2 μm.

The terms discussed above may be summed in quadrature to yield a standard uncertainty of about 12.2 μm in the length (for the 7.75 m length of combination 1). This uncertainty is the ‘noise’ and is to be contrasted against the ‘signal’ which is the error in the length (residual from mean length) due to the second order scale error of the encoder. The length error due to second order harmonic scale error is within ±65 μm as pointed out earlier.

We perform 18 such length measurements from which we extract 2 parameters (*a*_2_ and *b*_2_—the coefficients of the second order harmonic). A Monte Carlo Simulation yields a standard uncertainty of 0.4 μrad on the parameters *a*_2_ and *b*_2_ resulting in a 0.4 μrad uncertainty on an arbitrary point in the error map itself (the uncertainty in the scale error map *e*(*ϕ*) at any azimuthal position *ϕ*, where *e*(*ϕ*) = *a*_2_cos(2*ϕ*) + *b*_2_sin(2*ϕ*). Other simulations suggest that the standard uncertainty on the parameters remains 0.4 μrad if an attempt is made to estimate both the 2nd and 4th orders from the 18 length measurements, and the standard uncertainty on an arbitrary point in the error map (uncertainty on *e*(*ϕ*) = *a*_2_cos(2*ϕ*)+*b*_2_sin(2*ϕ*)+*a*_4_cos(4*ϕ*)+*b*_4_sin(4*ϕ*)) is about 0.5 μrad.

The standard uncertainty in the length for combination 2 (*D* = 4.9 m, *L* = 5.4 m; the range to targets *R* is 5.6 m and the half angle *θ* subtended by the length is 29°) can be calculated in a manner similar to that described above, and is 9 μm. Again, we distinguish between this ‘noise’ and the ‘signal’, where the ‘signal’ is the error in the length (residual from mean) due to second order harmonic scale error, which is within ±43 μm. The standard uncertainty in the two parameters calculated from 18 length measurements (*a*_2_ and *b*_2_—the coefficients of the second order harmonic) is about 0.4 μrad and there is also a 0.4 μrad uncertainty on an arbitrary point in the error map itself.

The maximum signal-to-noise ratio for combination 1 (*D* = 7 m, *L* = 7.75 m) is 65/12.2 = 5.3, and is marginally better than the maximum signal-to-noise ratio for combination 2 (*D* = 4.9 m, *L* = 5.4 m) which is 43/9 = 4.7. This does seem to suggest that a longer length placed farther away is more suitable for this test, but practical limitations such as space constraints will most likely limit the size of the length and its placement. A 7.75 m long length placed 7 m away does appear to be adequate in mapping out second order errors.

## 8. Summary

A tracker is designed to measure large lengths at long distances with a high degree of accuracy. The 7.75 m long length placed 7 m away that was used in this study is a fairly representative measurement for a tracker. Older trackers from two different manufacturers that we tested produced length errors as large as 65 μm due to second order scale error. The maximum permissible error for this measurement is approximately 120 μm based on a typical manufacturer’s specifications, without considering temperature effects. The second order scale error is a significant fraction of the MPE.

The method described in this paper provides a simple technique for estimating scale errors in the horizontal angle encoder of a laser tracker. It does not require expensive instrumentation or even a calibrated artifact. It is quick and easy to perform and can be realized in an environment with reasonable degree of stability.

We did correct the horizontal angle encoder error map of two trackers which were from two different manufacturers. The corrections were based on the horizontal length tests as described in this paper and, as expected, subsequent length measurement system tests revealed the absence of any second order terms. The length errors reduced from a maximum of about 65 μm before correction to less than 25 μm after correction. Newer trackers from two different manufacturers did not reveal this second order error; neither did an old tracker from the third manufacture.
